# Human 3D Pose Estimation with a Tilting Camera for Social Mobile Robot Interaction

**DOI:** 10.3390/s19224943

**Published:** 2019-11-13

**Authors:** Mercedes Garcia-Salguero, Javier Gonzalez-Jimenez, Francisco-Angel Moreno

**Affiliations:** Machine Perception and Intelligent Robotics Group (MAPIR), Dept. of System Engineering and Automation Biomedical Research Institute of Malaga (IBIMA), University of Malaga, 29071 Málaga, Spain; javiergonzalez@uma.es (J.G.-J.); famoreno@uma.es (F.-A.M.)

**Keywords:** human body pose estimation, 3D computer vision, camera pose calibration, human–robot interaction, OpenPose, RGB-D cameras

## Abstract

Human–Robot interaction represents a cornerstone of mobile robotics, especially within the field of social robots. In this context, user localization becomes of crucial importance for the interaction. This work investigates the capabilities of wide field-of-view RGB cameras to estimate the 3D position and orientation (i.e., the *pose*) of a user in the environment. For that, we employ a social robot endowed with a fish-eye camera hosted in a tilting head and develop two complementary approaches: (1) a fast method relying on a single image that estimates the user pose from the detection of their feet and does not require either the robot or the user to remain static during the reconstruction; and (2) a method that takes some views of the scene while the camera is being tilted and does not need the feet to be visible. Due to the particular setup of the tilting camera, special equations for 3D reconstruction have been developed. In both approaches, a CNN-based skeleton detector (OpenPose) is employed to identify humans within the image. A set of experiments with real data validate our two proposed methods, yielding similar results than commercial RGB-D cameras while surpassing them in terms of coverage of the scene (wider FoV and longer range) and robustness to light conditions.

## 1. Introduction

Human–Robot Interaction (HRI) is a key problem when a mobile robot has to share a common environment with humans. HRI has been widely investigated and developed during the last decades, producing several works that have mainly focused on proximate and direct interactions [1]. In this context, the desirable robot capabilities include recognizing gestures [2], understanding non-verbal cues during conversations [3] or approaching people in a natural manner [4,5]. Before starting any of these interaction tasks, the robot needs to locate the human with respect to itself, i.e., the relative position and orientation between both of them must be determined. This becomes a challenging chore due to several factors, including the presence of multiple humans, occlusions, person-alike objects (e.g., photos), and the inherent complexity and diverse nature of the human body. Moreover, the limited computational and sensing resources on-board, as well as the requirements of operating in real-time and in motion, aggravate the problem.

**Figure 1:**
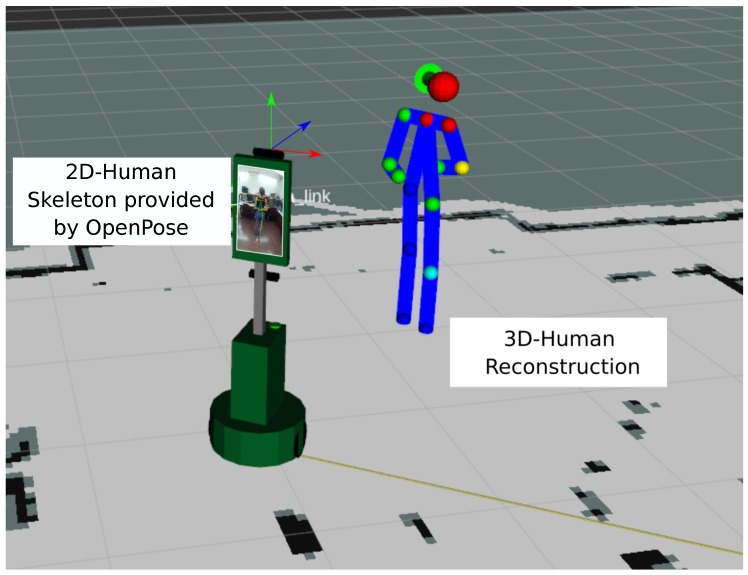
The people detector model identifies the 2D human joints within an image. The proposed methods reconstruct the human skeleton in 3D-space (*human body pose*) and provides a complete *human pose* (position and orientation).

It is important to clarify that, following the conventional nomenclature in robotics, we employ the term *pose* to refer to the *position* and *orientation* of a coordinate system (associated to a person, for example). Besides, the expression *human body pose* will denote in this work the estimated 3D coordinates of the joints belonging to a human body. In turn, the 2D projection onto the images of these coordinates will be denominated by the human *skeleton*, while the term *posture* will specify if the user is lying down on the floor (*horizontal* posture) or standing up (*vertical* posture). As an example of these concepts, Figure 1 shows a 2D human skeleton extracted by OpenPose displayed on the robot’s screen, and a 3D human body pose reconstruction within a representation of the environment in front of the robot.

Previous works have addressed the human pose and human body pose estimation with different methods and sensors. A common approach is to rely on RGB-D cameras, which simultaneously provide depth and visual information [6,7] at a low-cost. However, RGB-D cameras suffer from a set of issues that limit their applicability in real scenes, namely, a narrow field-of-view (FoV) (usually below 60 degrees), a short range of operation (typically under 3.5 m), and unreliable performance under adverse lighting conditions (particularly sunlight). Although the FoV limitation can be mitigated by mounting several cameras on the robot, the infrared patterns they project on the scene may interfere with each other [8]. To alleviate this, special attention has to be paid to their spatial arrangement, even setting up special hardware configurations [9]. All these limitations eventually preclude their usage for human detection in non-controlled situations (as, for instance, mobile robot navigation within homes), and might confine them to applications that restrict the human presence to a specific, relatively small area.

**Figure 2:**
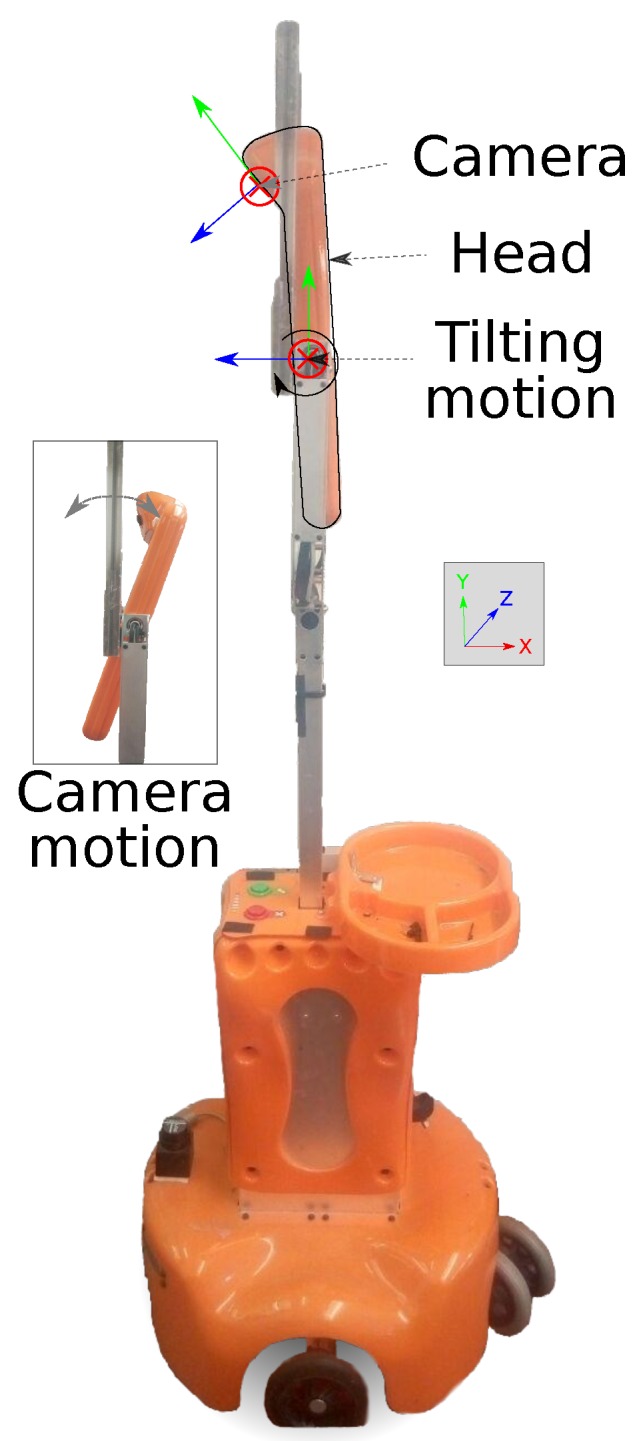
*Giraff* service mobile robot endowed with a tilting camera employed during this work. The maximum, positive tilt angle is depicted: it can be seen how the principal translation occurs along the *z*-axis of the camera frame.

In this paper, we investigate the use of a wide FoV RGB camera for determining the 3D position of the joints of a human body (*human body pose*), and estimating from them the human 3D position and orientation (*human pose*). Due to relying on RGB images, our proposed system overcomes all the above-mentioned issues presented by RGB-D cameras, and provides a mobile robot with the ability of, first, robustly detecting a person while moving, and then determining their pose within the environment (refer to Figure 1). This two-stage pipeline allows the robot to properly generate a suitable navigation path towards the users as well as to establish a social interaction with them. Furthermore, our proposal is able to estimate the user location even if they are either standing up or lying on the floor, which is a crucial situation in certain robotic applications such as robots assisting elders at home. Specifically, we employed the *Giraff* mobile robot [10], which is equipped with a controlled tilting head, as shown in Figure 2. This allowed us to benefit from some of the stereo vision advantages.

As explained below, employing a moving camera demands a precise estimation of the sequence of camera poses with respect to the robot. Given the particular motion that the on-board camera undergoes, dissimilar to the standard configuration of a fixed attachment to the robotic platform, a specific section for camera extrinsic calibration is included in this paper.

In this work, the human detection task is carried out by a well-known approach based on Deep Convolutional Neural Network (DCNN) called *OpenPose* [11], which is able to detect multiple 2D humans within an image. Please note that *OpenPose* only provides 2D image coordinates, i.e., the skeleton of the detected humans, and not the 3D position and orientation of the detected person, even though the name includes the term *pose*. Built upon this detection system, we propose two complementary methods for the reconstruction of the human 3D pose:

- ***Single-View***. The first method requires only a single image of the scene (provided that the human feet are visible) to reconstruct a simplified, planar version of the detected human’s skeleton. Since it is fast, this method performs well even if the robot and/or users are moving, allowing its use during normal robot navigation.
- ***Multi-View***. This second approach is more precise and can be applied with the user in any arbitrary bodily position. It uses several (at least two) views of the scene taken from different angles of the tilting robot head so that both the user and the robot must remain reasonably still during the acquisition time (≈2 s). It is important to highlight that, in this case, and due to the particular camera setup and motion, common epipolar constraints for stereo vision are not applicable. Thus, this paper also contributes with an implementation of specific 3D reconstruction equations, as well as with the exploitation of the body structure to improve the obtained results.

These two methods were validated both in a static configuration and with moving users in an indoor environment (see Section 5), yielding accurate results comparable to those that can be directly obtained with RGB-D-based cameras, while significantly extending the applicability, versatility and robustness of the detection process thanks to the above-mentioned advantages of RGB cameras in terms of FoV, maximum range and lighting conditions.

## 2. Related Work

Many previous works have addressed human pose and human body pose reconstruction based on different strategies. In this section, we summarize the existing image-based approaches, categorizing them as: (i) probabilistic approaches; (ii) direct user-supplied information; (iii) non-euclidean representations; (iv) motion-based approaches; and (v) ConvNet architectures and training methods. The work in [12] provides a comprehensive collection of methods for human body pose estimation based on monocular images.

**Probabilistic approaches** rely on the analysis of a sequence of images. Thus, in [13], a hybrid Monte Carlo filter is employed to estimate the 3D shape of a moving person from 2D image markers. A three-stage approach based on previous works and a Bayesian formulation is proposed in [14] for a robust 3D pose tracking and recovery. However, limited on-board resources and restrictive time requirements preclude their usage on mobile robots.

Other works employ **prior and/or user-supplied information** about the person in order to estimate or initialize the body pose. The work in [15], for example, recovers the 3D human body pose by considering the foreshortening of the limbs, whose lengths are given as inputs for the algorithm. In turn, in [16], these limb lengths are predicted based on the known human height and a gender-height indexed database. The initial pose is created based on user-supplied joints observations and further optimized with a parametric 3D body model. The proposal in [17] requires the user to manually label the 2D joint locations, estimating the sparse model for these observations from known limb proportions. These specifications about physical attributes limit the generalization and unsupervised utilization of the systems. Furthermore, scenarios with more than one person are not considered, which is a situation that a mobile robot regularly has to deal with.

**Embedded spaces** that encode the human skeleton structure have been also proposed to recover an anatomically feasible human body. The works in [18,19], for instance, model shape deformations as non-Euclidean representations, adding the latter the viewpoint to the model. Although the obtained human bodies fulfill kinematic and anatomic constraints, these approaches do not provide the 3D pose with respect to the camera, but only the body configuration and deformation, which is not the main scope of our project.

There exists another set of approaches that employs and computes the **human motion** as part of body estimation. For instance, Urtasun et al. [20] incorporated a strong motion model to generate a full 3D reconstruction, for both monocular and multi-view tracking, although the number of examples needed to create the database employed to build the model becomes the main limitation. In turn, the work in [21] proposes an optical flow approach to recover human motion from monocular images and a human body model. The motion is estimated by minimizing the error between the computed flow and an artificial flow renderer. Although the authors obtained appealing results, motion-based approaches are focused on human bodies that must be estimated through time, rarely providing the pose with respect to the camera, a major goal in our project. Additionally, temporal information is avoided in this work in order to endow the system with time-independent properties.

Finally, in recent years, we are witnessing amazing results in the computation of human skeletons and body 3D reconstructions upon techniques based on ***Deep Neural Networks*** (*DNN*) In [22], for example, a multi-stage DNN-based regression to Cartesian joints coordinates is formulated and tested, while, in [23], a hybrid architecture for monocular images that is based on a *Convolutional Neural Network* and a Markov Random Field inspired by a Spatial-Model is proposed. However, since these methods require a large amount of training data, they are usually confined to constrained environments and situations [24]. On the other hand, the work in [25] combines 2D images and 3D motion data in order to generate a large training set which allows to train an end-to-end CNN 3D pose classifier.

Following a different strategy, the method of Martinez et al. [26] decouples the 3D pose estimation into two stages: a 2D pose estimation followed by a 3D pose estimation from the 2D joint detection. Their work focuses on the second stage and shows that inferring 3D joints from 2D projections can be solved with a relatively low error rate. A similar approach to our proposal is described in [27], where the authors presented a **sparse** multi-view system where the human body joint detection is also carried out by *OpenPose*. For that, several images (at least three) taken from very different points of views of the scene and a two-stage assembling method that selects the correct 3D human body pose from thousands of pose seeds combined according to joint semantic restrictions are employed. Unfortunately, the total running time per estimation is reported to be 14 s. In comparison to our method, the cameras’ layout for this approach becomes unsuitable for many situations such as, for example, applications involving mobile robots and/or outdoor environments (at least without any additional hardware). Besides, the excessive running time precludes its employment in real time scenarios.

Although previous works have obtained interesting results, the elevated computational cost (e.g., workstations with up to 12 CPU cores are employed in some of them) excludes a mobile robot (with usually 1 CPU) or even a simple distributed system as the platform target for the implementation. Additionally, multi-view systems, such as the one in [27], often require many different and sparse views, also avoiding their usage with mobile robots, which are usually endowed with only a few cameras (or even just one) with similar positions on the robot.

In contrast to the works described in this section, our proposed estimation system is a lightweight method whose major computational cost is the inference of the people detector model, being suitable for the embedded computer in the robot or a simple distributed system. Besides, no prior knowledge about the humans (or their motion), or any user-supplied information is required, allowing both the automation and unsupervised use of the system. Furthermore, the estimation of multiple humans’ poses at once is also possible, even if they are lying on the floor. Finally, our system is endowed with time-independent properties, reducing the delay for the reconstruction and allowing the robot to start and finish the estimation at any time.

## 3. Camera Extrinsic Calibration

This section first briefly describes the employed robotic platform and then introduces the proposed camera extrinsic calibration method.

### 3.1. Robotic Platform

The service robot *Giraff* [10] (Figure 2) has been specifically designed for HRI with elders and progressively developed within a sequence of the European projects AAL [28], GiraffPlus [29] and MoveCare [30]. The current robot’s hardware consists of a monitored wheeled platform with two casters and two independent wheels, an on-board computer and a tilt-adjustable *head* (providing a pitch rotation), where a 5 Mpx fish-eye webcam is placed. A 2D laser rangefinder (Hokuyo URG-04LX-UG01) was added to enhance its localization and autonomous navigation capabilities.

Our work exploits one of the possible applications of the tilting camera, whose driver allows us to set the inclination angle within a certain range. Feedback from the motor controllers is not available; hence, there is no guarantee that its final position is reliably known. Therefore, we implement a refinement and confirmation stage that employs a set of features detected on the robot platform itself, as will be explained next. Figure 2 also depicts the *Giraff* robot with the tilting head and the camera, where their associated reference systems are marked, and the position of the camera for the maximum positive tilt angle is shown.

### 3.2. Camera Pose Estimation

To determine the user’s position with respect to the robot, we first need to estimate the pose (i.e., position and orientation) of the camera with respect to the robotic platform. For that, we compute the different camera poses (represented as rigid transforms in SE(3), Special Euclidean Group) with respect to a fixed reference frame (called here *floor frame*) that lies on the floor or on a parallel plane *XZ* with the positive *y*-axis pointing upwards. Although there are infinite reference systems that fulfill these constraints, any of them provide us with the needed information:

- A rotation transformation **R** that aligns the camera’s *y*-axis with the *y*-axis of the *floor frame*, so that the camera’s *XZ* plane becomes parallel to its correspondent in the *floor frame*.
- The *y*-coordinate of the camera frame with respect to the *floor plane*, that is, the distance (or height) from the floor to the camera center.

From this information, we build a rigid body transformation P that can be expressed as the 4×4 matrix in Equation (1), where R∈SO(3) and $t\in R^{3}$:

(1) $$P=\left(\begin{array}{cc} R & -Rt\\ 0 & 1 \end{array}\right)\in SE\left(3\right)$$

Thus, to estimate a general *i*th camera pose ${\hat{P}}_{i}=\left[R_{i}|t_{i}\right]$, given a set of known 3D points $\left\{X_{k}\right\}$ with respect to the *floor frame* and their correspondent 2D projections $\left\{z_{\mathrm{ki}}\right\}$ measured on a single image, we minimize the energy function in Equation (2) with respect to ${\hat{P}}_{i}$ using the conjugate gradient optimization method, with the Huber loss function (ρ) in an approximation of the SE(3) as the product $SO\left(3\right)\;\times \;R^{3}$, which allows avoiding the singularity related to rotations with Euler angles [31].

(2) $$\phi \left({\hat{P}}_{i}\right)=\frac{1}{2}\sum_{k}\rho ({\left\|{\tilde{z}}_{\mathrm{ki}}-{\hat{P}}_{i}{\tilde{X}}_{k}\right\|}^{2})$$

**Figure 3:**
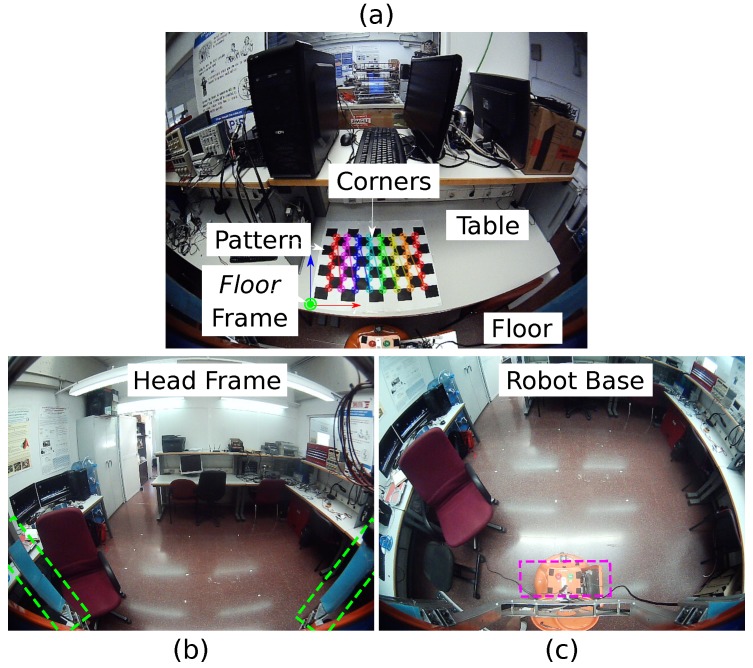
(**a**) Chessboard pattern employed for the camera extrinsic calibration and *floor* frame in which the camera pose is defined; and (**b**,**c**) set of detected features that allows retrieving the camera pose during normal operation: (**b**) *head* and (**c**) *base*.

A chessboard pattern with known size lying on a table (or on a parallel plane to the floor), like the one shown in Figure 3a, is employed to generate the 3D–2D correspondences. We set one of its corners as the origin of the *floor frame* and we define the columns and rows of the pattern as the directions of the x- and z-axes of this reference frame, respectively. The corners of the chessboard are then identified and employed as features for performing camera calibration. This process is repeated for a discrete set of tilting angles $\left\{\psi_{i}\right\}$ that covers the complete range the motor driver allows, storing the obtained ${\hat{P}}_{i}$ matrices so that they can be later retrieved, leading to a map $\left\{\psi_{i}\right\}⟶\left\{{\hat{P}}_{i}\right\}$.

Nevertheless, this mapping is not enough since the positioning of the camera by the motor driver is not reliable. Therefore, we also extract a set of visual features $F_{i}$ from the captured image at each camera pose that is then employed to retrieve the transformation matrix ${\hat{P}}_{i}$, extending in this way the above-mentioned mapping so that $\left\{\psi_{i},F_{i}\right\}⟶\left\{{\hat{P}}_{i}\right\}$.

To extract these visual features, we make use of the robot structure by finding two distinctive sets of attributes:

- ***Base***: The image coordinates of the robot’s action buttons on the base are stored as the visual feature vector for each camera pose. This is employed for positive camera rotations, since only the robot base is visible in the image, as shown in Figure 3c.
- ***Head Frame***: The head frame appearance is highly changeable during the camera movements, which precludes employing a fixed template as before in order to extract the features. For this reason, we employ the angles between the head frame and the image borders to build the feature vector. This is done for negative rotations (see Figure 3b).

During normal operation, we compute $F_{i}$ in five consecutive images in order to increase their accuracy. Additionally, the tilt angle corresponding to the recovered camera pose is compared with the tilt angle that was commanded to the head motor, rejecting the retrieved pose if the error between these two values is greater than a certain threshold. In this case, the tilt command is re-sent and the feature detection algorithm is run again. This process is repeated until the camera pose is successfully retrieved or a fixed, small number of iterations is reached. This way, we are able to detect both incorrect camera motions and unreliable feature detection.

## 4. Human Body Pose Estimation

In this work, we propose a human body pose estimation algorithm that follows two consecutive stages: (1) an image-based human body identification; and (2) a 3D body part pose estimation. For the first stage, we employ *OpenPose* [11], an efficient method for multi-person 2D pose estimation. It infers the human figure in the image and returns a set of keypoints, joints or *body parts*, which conform to a simplified 2D human skeleton in the image, along with a confidence value for each part.

The second stage aims to position these keypoints in the space, obtaining a simplified 3D human body representation. Two approaches are presented for this second task, namely *Single-View* (Section 4.1) and *Multi-View* (Section 4.2). As further described below, the former requires just an image to generate an approximation of the human body in 3D, increasing the speed of the estimation and allowing both the human and the robot to be in motion during its process. As a constraint, this approach needs the human’s feet to be visible in the image. The latter, in turn, requires at least two views of the scene and the human to remain static, but produces results without any prior restriction on the human’s bodily position. It is important to highlight, however, that the aim of this work is to obtain an accurate estimation of the human pose but not a precise 3D reconstruction of the human skeleton.

### 4.1. Single-View Estimation

This method estimates the whole human body pose in the space employing a single image and the body parts extracted from it. Our approach is based on the mild assumption that the human feet (parts identified by *OpenPose*) are in contact with the floor, projecting and simplifying the human body as a plane. We must add that this method is valid even when the human is lying on the floor, making our work extensible to these situations. However, the feet must have been detected to be able to apply this approach while other, more complex human bodily positions such as sitting or bending generate worse reconstructions. These situations, however, can be handled with our *Multi-View* approach.

The proposed algorithm is composed of four stages: (i) feet position estimation; (ii) human posture identification, which simplifies the estimation of the 3D body pose; (iii) body parts triangulation; starting from the feet; and (iv) human orientation estimation.

**Feet Position Estimation**. Assuming that the feet are in contact with the floor, from the homogeneous coordinates of the feet within the camera sensor ${\tilde{q}}_{i}=\left[q_{xi}:q_{yi}:q_{zi}\right]$, and the perpendicular distance from the floor to the camera frame $H_{c}$, we can triangulate their 3D position $Q_{i}$ with respect to the camera reference system by applying:

(3) $$Q_{i}=H_{c}\frac{1}{q_{yi}}{\tilde{q}}_{i}$$

To obtain the value for $H_{c}$, we first retrieve the camera pose ${\hat{P}}_{i}=\left[R_{i}|t_{i}\right]$, as explained in Section 3.2. The second component of the translation vector $t_{i}$ provide us the value of $H_{c}$, while the rotation matrix $R_{i}$ is employed in the following stages.

**Human Posture Identification**. After computing the feet 3D positions $Q_{i}$, we infer the human’s posture (horizontal or vertical) with respect to the camera frame by studying the dispersion of the detected joints coordinates (except the arms, since they do not provide with useful information about the human posture) along the xi and y-axes of the camera frame. The value of the maximum dispersion informs us about the dominant dimension, and thus, the human posture with respect to the camera frame.

**3D Body Parts Triangulation**. Now, from this information, we consider two situations: the person is standing up (vertical posture) or lying down (horizontal posture). For the former, we simplify the human body by projecting it over the plane $Z=Z_{0}$, with $Z_{0}$ being a constant value computed as the mean of the feet Z-coordinates. The 3D body parts $Q_{i}$ are then computed employing their correspondent image observations ${\tilde{q}}_{i}$ applying Equation (4). In turn, if the user has a horizontal orientation, we simplify the human body by projecting it over the floor plane Y=0. Thus, the 3D body parts can be computed by applying Equation (3). Note that this reconstruction does not depend on the feet identification, removing this requirement in such situations.

(4) $$Q_{i}=Z_{0}\frac{1}{q_{zi}}{\tilde{q}}_{i}$$

Finally, it has to be noted that, to employ the former Equations (3) and (4), the body parts observations ${\tilde{q}}_{i}$ need to be referred to a coordinate system whose XZ plane is parallel to the floor. At this point, the rotation matrix $R_{i}$ from the camera pose (refer to Section 3.2) provides us with the needed transformation.

**Human Orientation Estimation**. Finally, to estimate the orientation, we project the human torso over the floor (Y=0), computing the angle between the shoulders and the neck projections. The errors in the detection and projection of the body parts, missing joints, and incoherent results are filtered out by employing the detected hip joints of the human, the torso appearance within the image and other physical human constraints. Additionally, these considerations allow us to infer the 3D position of a missing shoulder, if needed, and to improve the human reconstruction by imposing the computed orientation to the upper-body joints.

This algorithm is also employed to estimate the human orientation in the *Multi-View* method, since it does not depend on either the human feet or their identification.

### 4.2. Multi-View Estimation

This second method is able to locate the human even when the feet have not been detected and without any prior information about any body part or simplification about the human body. The algorithm requires: (i) more than one view of the scene; and (ii) the person to remain static at the same position while the images are being captured. To obtain the different views with only one camera, this is moved, as shown in Figure 2.

Due to the inherent noise in the joints identified by *OpenPose*, we detect them in a set of images (which are only employed for the human detection, not for the body reconstruction) and weight each joint position by its associated confidence value provided by the detector. This leads us to a better estimation of the body parts position in the image. These joints are then matched within another set of images captured for body reconstruction through the procedure described in this section.

To estimate the correspondences and obtain the 3D associated points, we rely on the cameras’ relative pose through the *epipolar geometry*. The ideal stereo camera configurations (e.g., [32,33,34]) have baselines where the primary component is in either the *x*- or *y*-axis direction. However, the arc-like movement of our tilting camera generates a translation vector with the *z*-axis as the predominant direction, and also with a rotation in the *pitch* angle, therefore positioning the epipoles inside both image planes. This configuration impedes the usage of the well-known and optimized algorithms designed to find stereo correspondences and to estimate their 3D projections from their observations [35,36]. To overcome this issue, we adapt and implement the standard procedure to our scenario following three stages: (i) image rectification; (ii) correspondence problem; and (iii) triangulation.

**Image Rectification**. The rotation and perspective change between two images in our system hinders the correspondence problem. The usual procedure consists of applying a planar rectification to both images in order to translate the epipoles to the infinity in the X or Y direction, leading to horizontal epipolar lines. Since this method only works when the original epipoles are further away from the image planes, it is not suitable for our configuration. Therefore, we implement and employ a rectification method based on two steps:

1. **Initial rotation**. Once we have a pair of images taken from two different camera poses, we first transform the second image by applying the rotation matrix $R_{i}$ between the cameras, aligning this way both reference systems. This matrix generates a linear transformation through the expression: $H_{i}={\mathrm{KR}}_{i}K^{-1}$, where K is the camera’s intrinsic calibration matrix, which can be directly applied to the image. However, this alignment presents small errors that have to be corrected by finding a second (residual) rotation that completely matches the axes.
2. **Residual rotation**. The residual rotation matrix $R_{R}$ aligns both epipoles *e* and $e^{\prime }$, so that $R_{R}e^{\prime }=e$ [32,37], and it is computed based on the homogeneous coordinates of the epipoles as:
  (5) $$R_{R}=I+{\left[e^{\prime }\times e\right]}_{x}+{\left[e^{\prime }\times e\right]}_{x}^{2}\frac{1-e^{\prime }{}^{T}e}{{\left\|e^{\prime }\times e\right\|}_{2}^{2}},$$
  where **I** denotes the square identity matrix of size 3 and ${\left[▪\right]}_{x}$ stands for the skew-symmetric cross-product matrix equivalent for ▪ [32]. Again, a new linear transformation $H_{R_{R}}$ is applied to the image. Note that Equation (5) is the Rodrigues formula [32] for the non-unit rotation vector $\left[e^{\prime }\times e\right]$ with rotation angle $e^{\prime }{}^{T}e$.

**Figure 4:**
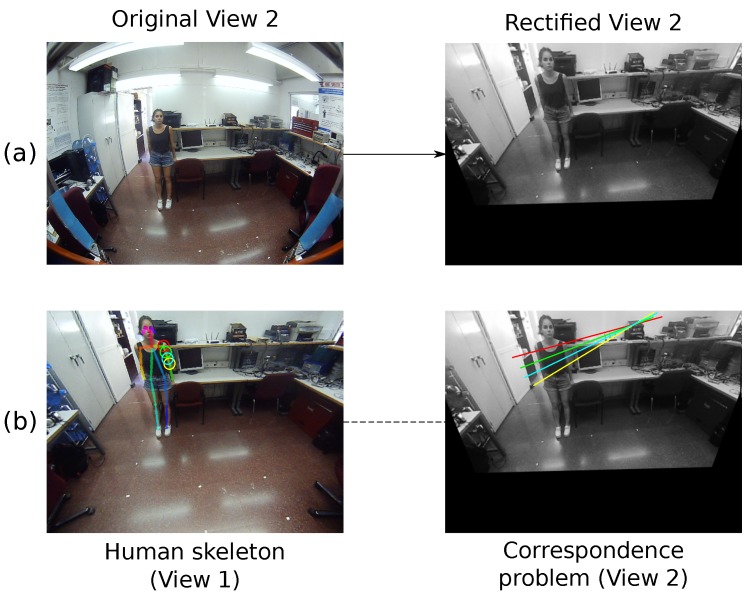
(**a**) The original second view is rectified by applying two consecutive rotations. (**b**) The joints (circles) belonging to the human skeleton provided by *OpenPose* in the first view are searched within the second one applying epipolar constraints (lines).

Figure 4a shows an example of this process (left) before and (right) after the image rectification.

Additionally, based on the epipole coordinates and the principal translation $b_{z}$ (known from the cameras’ poses), we re-refine the baseline as: $b={\left[b_{x},b_{y},b_{z}\right]}^{T}=K^{-1}e=K^{-1}e^{\prime }$. This calculation allows us to obtain a baseline that is coherent with the appearance of the images.

**Correspondence Search**: Once the images are rectified, the detected joints are matched between views by employing the Normal Cross-Correlation (NCC) as a measurement of their similarity, introducing a minimum threshold ξ=0.9 to ensure correct correspondences, and applying the epipolar constraints associated to the epilines. Since the epipoles lay within the image planes, epilines are orientated [38]. Thereby, matching points must be disposed on the same zone of the epiline in order to avoid 3D-reconstructed points with negative depth. This constraint reduces the number of possible corresponding candidates as well as the computation time.

However, the corresponding points for the humans’ joints are not easy to localize and the matching process usually fails, losing track of the joint and, hence, its reconstruction. Therefore, to increase the number of observable points, we include the limbs in the process. A limb is defined as the solid rigid part linking two joints, which can be simplified by a 3D line with an associated 2D projected line in the image. In this sense, a human is depicted as a set of articulated limbs, forming a *skeleton* in the image. Given two observations corresponding to two joints belonging to the same limb, all the points from one observation to the other that follow the connection line between them are considered as observations of the limb’s points. An example of this can be found in Figure 4b (left), where the detected *skeleton* is represented along with a set of points belonging to a certain limb, while the right image shows their corresponding epipolar lines.

Furthermore, given two connected limbs, their intersection point corresponds to the joint, both in 3D and in the image. With this approach, not only we increase the number of available observations, but we also recover a missing joint between two limbs by intersecting them. To decouple possible errors, we search for each correspondence independently using RANSAC to compute the associated line equation and to avoid outliers. Additionally, we estimate the 2D intersection point **q** for a set L of *n* limbs projections ($l_{i}$), i.e., for the set $L={\left\{l\right\}}_{i=1,\ldots ,n}$ in order to constrain the subsequent 3D reconstruction, as explained next.

Due to detection errors, a single intersection point does not exist. Thus, we compute the closest point following a least-squares approach by applying Equation (6) [32], where each limb projection $l_{i}$ has been expressed as its direction vector ${\hat{v}}_{i}$ and a point belonging to the limb $p_{i}$.

(6) $$q={\left(\sum_{l_{i}=\left({\hat{v}}_{i},p_{i}\right)\in L}\left(I-{\hat{v}}_{i}{\hat{v}}_{i}^{T}\right)\right)}^{-1}\left(\sum_{l_{i}=\left({\hat{v}}_{i},p_{i}\right)\in L}\left(I-{\hat{v}}_{i}{\hat{v}}_{i}^{T}\right)p_{i}\right)$$

**Triangulation**: Once the corresponding points have been found, the 3D point associated with these observations can be triangulated. Once again, the standard equations derived for ideal configurations cannot be employed and they require a modification to deal with a more general camera configuration where the three baselines are not negligible (i.e., $b_{x}\neq 0,b_{y}\neq 0$ and $b_{z}\neq 0$), leading to the following expression for the 3D point $Q={\left[X,Y,Z\right]}^{T}$:

(7) $${\left[X,Y,Z\right]}^{T}={\left[xZ,yZ,\frac{bx+by-bz(x^{\prime }+y^{\prime })}{\left(x-x^{\prime }\right)+\left(y-y^{\prime }\right)}\right]}^{T},$$

where $x={\left[x:y:1\right]}^{T}$ and $x^{\prime }={\left[x^{\prime }:y^{\prime }:1\right]}^{T}$ are the corresponding observations referred to the sensor planes in homogeneous coordinates for the first and second images, respectively.

The 3D points are then filtered out to ensure positive depth and results coherent with the scene as, for example, the points belonging to the same limb are forced to be aligned in space, following the associated 3D line. RANSAC is employed again to compute this 3D line. Finally, the 3D position of a joint is computed as the intersection between: (i) all the limbs that contain it; (ii) the ray associated with the corresponding observation identified by *OpenPose*; and (iii) the ray associated with the 2D intersection point obtained during the correspondence problem.

**Figure 5:**
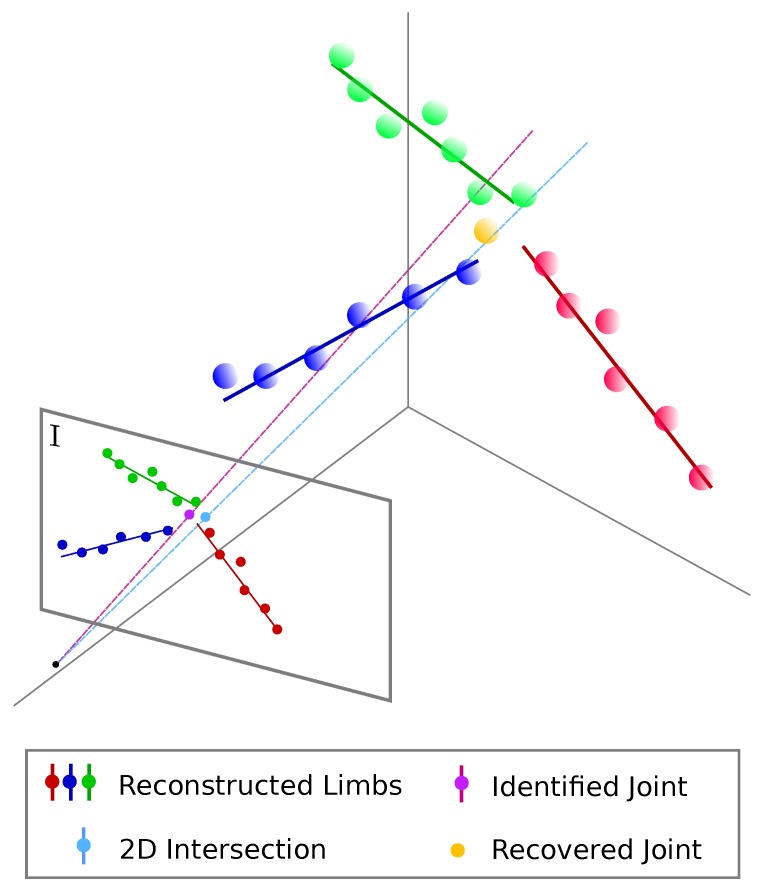
3D limbs in red, green and blue recovered based on their associated 3D points (same color). Their projection onto the image plane I allow us to compute the nearest point to all of them (cyan circle), which is employed along with the identified joint (purple circle) to retrieve the aforementioned 3D point.

This intersection problem is defined within the bounds imposed by the limbs and the identified joint coordinates. To illustrate this, refer to Figure 5, which represents three limbs (red, green and blue) obtained from the points belonging to them (points with the same color code) and their projection onto the image plane I. The purple circle depicts the identified joint by *OpenPose*, while the cyan one the 2D intersection of all the limbs projections computed during the correspondence problem. The 3D recovered joint (in orange) is the nearest point in terms of least square distance to all the lines. Finally, we impose human feasible dimensions by applying a median filter with a threshold of γ= 0.7 m to the recovered joints.

To increase the correct identification rate, more than two views are employed, since typical camera movements can leave out of view certain sections of the human body. The final results are merged into one single body estimation.

## 5. Experimental Validation

This section presents the validation tests that were carried out to assess the performance of our proposal. Please note that all our experiments employed real, unique data recorded with our tilting camera, as there are no publicly available datasets containing this kind of images. This prevented us from knowing the ground truth position for each body joint (without the employment of any additional technology). Therefore, in the presented experiments, we only estimated the *human pose* and not the full *human body pose*, although a qualitative body reconstruction (i.e., a *skeleton* with orientation) is also shown in the figures for a better understanding.

First, as presented in Section 5.1, we tested the repetitiveness of the camera pose retrieval system described in Section 3, while the rest of the sections are devoted to the human pose estimation algorithms and their performance. In these experiments, for the sake of clearness, we decoupled the tests for the position (Section 5.2) and for the orientation (Section 5.3). Concretely, as presented in Section 5.2, we estimated the position of a human through our two proposed methods, i.e., *Single-View* and *Multi-View* (Section 5.2.1 and Section 5.2.2, respectively), and compared their results against an RGB-D based 3D body tracking system. Besides, we applied our *Single-View* approach to the special case of humans lying on the floor, which is of particular interest for social robots operating in elder’s homes. Finally, as presented in Section 5.3, we validated the accuracy of the computed orientation in three different positions.

As the baseline for evaluating our proposal, we compared our systems with the results obtained with the SDK provided by the *Orbbec Astra* RGB-D camera, one of the state-of-the-art sensors for capturing depth information, which allows detecting and tracking the whole 3D human body in real time. This camera presents a FoV of 60° horizontally and 49.5° vertically, with a theoretical optimal depth range from 0.6 m to 5.0 m [39], as stated in the specifications provided by the manufacturer. In practice, however, human body detection is only reliable at a maximum distance of 3.5 m. Our tilting camera, in turn, is a 5 Mpx fish-eye RGB webcam with almost 180° of horizontal FoV. Due to its novelty (the SDK was released in 2018), just a few practical applications [40] explicitly employ this sensor. However, recently, the work in [41] proved through extensive experimentation that the *Orbbec Astra* camera performs similarly to the well-known and accurate *Microsoft Kinect* sensor [42] (indeed, they are reported to be interchangeable). The *Microsoft Kinect* camera, in contrast, has been widely tested in recent years in many applications, which have been reported elsewhere (e.g., [43,44,45]). Interestingly, and as an example of their accuracy in 3D human body estimation, *Microsoft Kinect* cameras have even been employed for physical rehabilitation, as described in MIRA [46,47].

### 5.1. Camera Retrieval

**Figure 6:**
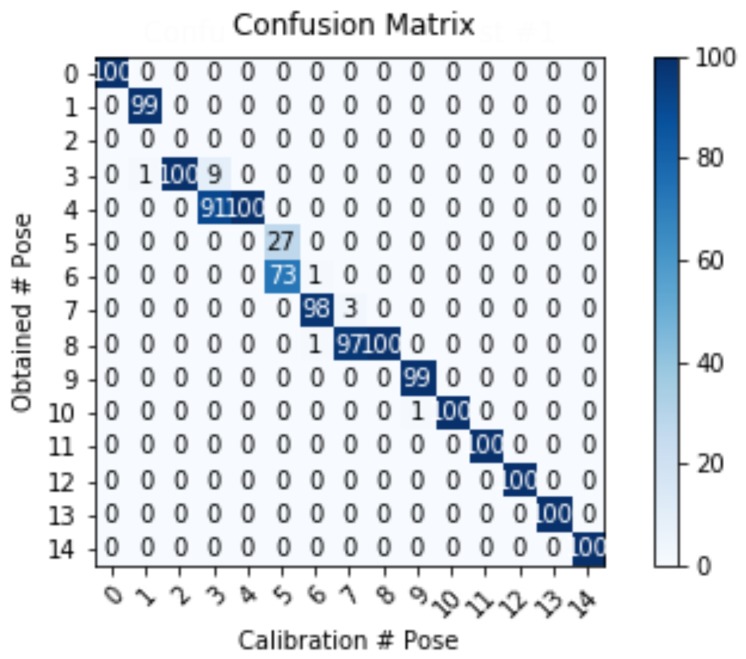
Fourteen gradually increasing and unknown poses were retrieved by our algorithm. The high number of coincidences reported reinforces the repetitiveness of the method.

The correct retrieval of the cameras’ poses based on the own robot platform visual features is essential for this work. To evaluate the repetitiveness of the algorithm, we positioned the camera in an arbitrary, unknown orientation and retrieve its pose 100 times. Figure 6 presents the confusion matrix for the estimated and the calibration poses (*Calibration # Pose*: 0–8 for positive tilt angles and 9–14 for negative). The tilt angles were chosen to increase gradually, hence the near diagonal aspect of the confusion matrix, while the high repetitiveness of the estimation is represented by the number of coincident recovered poses (>95 in most of the cases).

### 5.2. Position

In this section, we first compare the results in the position estimation of our *Single-View* method against the 3D human position estimation provided by an *Orbbec Astra* RGB-D camera, and test the performance of the proposed method with moving and lying humans (Section 5.2.1). In turn, Section 5.2.2 shows examples of 3D body reconstructions estimated with our *Multi-View* algorithm under challenging configurations, and the mean error for twenty different positions, as will be further described. Since the experimental setup is the same for both considered human positions, here we report the results yielded by the *Orbbec Astra* camera only in for the *Single-View* approach.

#### 5.2.1. Single-View Method

Three different tests were carried out to assess the *Single-View* method results for the position estimation, for both moving and still humans, keeping the robot static in order to simplify the validation. In *Test #1*, we compared the accuracy of the recovered human position with our method and the application provided by [39] for twenty discrete locations uniformly distributed within the overlapping cameras’ FoV. *Test #2*, in turn, was an experiment where a person is following a known, one-dimensional path. In *Test #3*, a similar experiment was carried out but with a rectangular path this time.

Note that, for these tests, and to compare both methods, we considered only the human position projected on the ground plane, i.e., without the height.

**Test #1**: In this experiment, we evaluated the error in twenty different, discrete and uniformly allocated positions, with the ground truth positions confined in a 1.6 × 1.2 m^2^ rectangle in front of the camera. We then compared the performance of our system with that of the *Body Tracking SDK*, provided with the *Orbbec Astra* camera, with its estimated pose referred to the same robot coordinate frame. The *tracking mode* of the SDK was employed during the tests to increase its detection rate in difficult conditions, e.g. when the human was not facing the camera.

**Figure 7:**
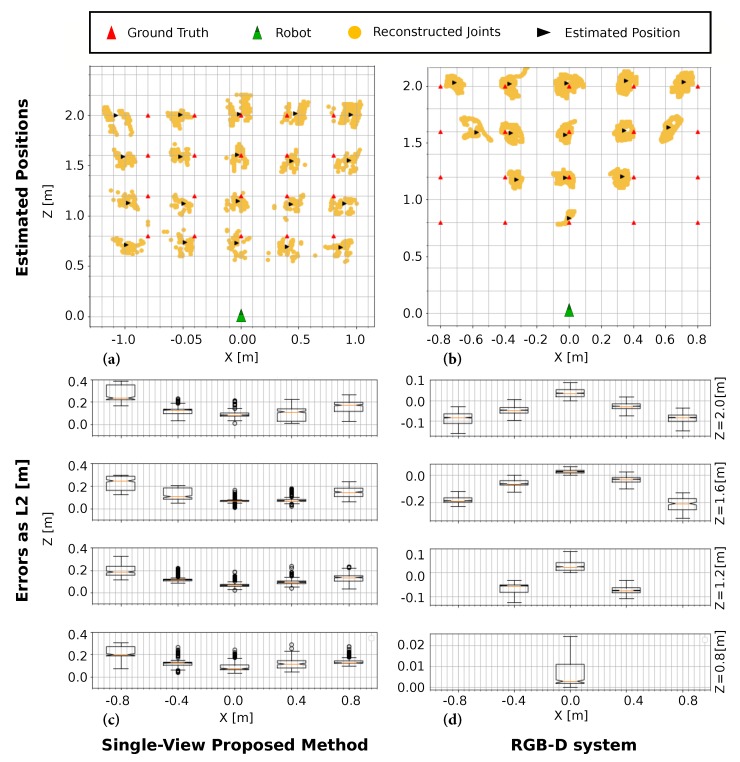
Test #1: (**a**,**b**) Estimated positions (black) for twenty ground truth points (red), along with the detected feet (orange). (**c**,**d**) The errors measured as L2-distances from the estimated user positions to their corresponding ground truth value.

Figure 7 depicts the obtained human positions for our method in (a) and the *Orbbec* system in (b), showing the corresponding errors in (c,d), measured as *L2-norm* from each foot to the corresponding ground truth position. It can be seen that, in our proposal, the error for the X-component increases outwards and radially, corresponding to the effect of a residual lens distortion, which is challenging to completely remove in *fish-eye* cameras [48], like the one placed on the robot. However, since this error is approximately independent on the depth value Z, we can model it as a quadratic equation on the X value by fitting the obtained errors, and include it as a bias in the estimation algorithm to correct it. On the other hand, the narrow FoV of the RGB-D camera is reflected in the right part of Figure 7. When the human is close to the robot (Z < 1.2 m), the estimation is confined into the center positions. Furthermore, even when the human is detected, lateral positions such as X ± 0.8 m present larger errors than their neighbours.

**Figure 8:**
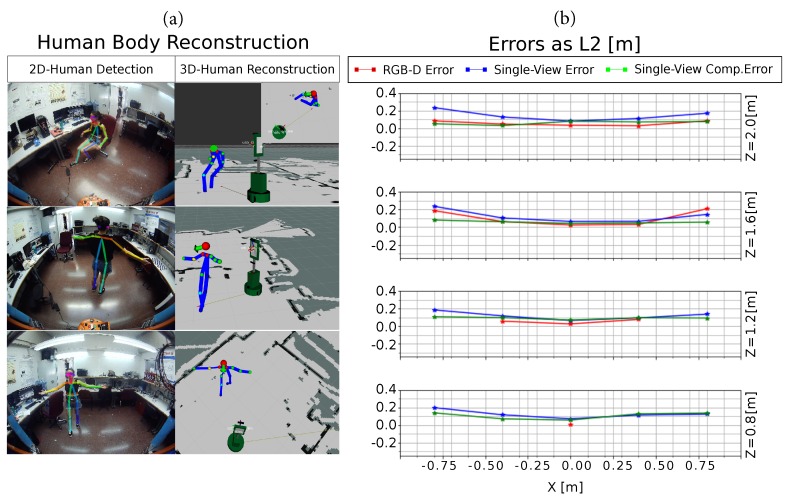
Test #1: (**a**) Three human body reconstructions obtained with our *Single-View* method; and (**b**) average errors for the twenty ground truth positions obtained with the RGB-D system (red) and our *Single-View* method, both original (blue) and compensated (green).

In turn, Figure 8a shows three different body reconstructions obtained with our proposed *Single-View* method. Note that the represented 3D bodies have been oriented according to the computed human orientation (as explained below), depicted as a green arrow in the figure, hence qualitatively increasing the faithfulness of the reconstruction. The plot in Figure 8b depicts the average error for the RGB-D method (red), our proposal (blue) and its compensated version by the above-mentioned parabolic fitting (green). It can be seen that our compensated method is able to rectify the error at the lateral positions, providing the best results in those locations. Our proposed system is able to estimate the human position in every established ground truth location, with similar or even lower errors than the RGB-D-based estimation, proving this way its suitability for human detection. Furthermore, the maximum depth range for both approaches was empirically estimated yielding that our proposed system can reconstruct a person 1.6 m tall up to a distance of 5 m, hence increasing the depth range in 1.5 m with respect to the RGB-D-based system.

**Figure 9:**
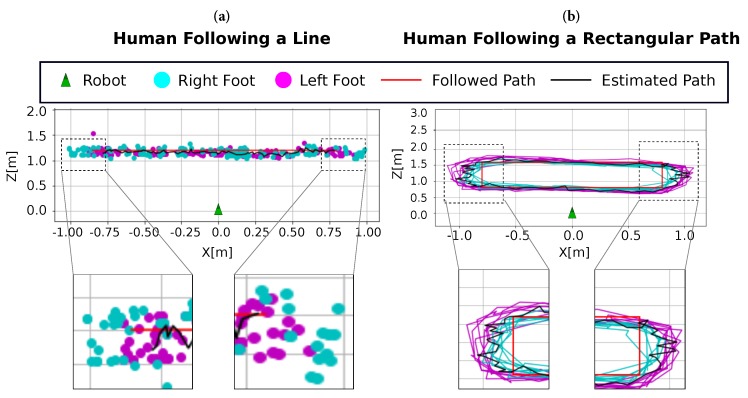
(**a**) Test #2: Linear path test and details of the trajectory endpoints. (**b**) Test # 3: Rectangular path test and details of the trajectory laterals. For both tests, we show: (top) the estimated poses for both feet (cyan and magenta) with respect to the robot position (green) and the followed path (red); and (bottom) detail of the path endpoints. The estimated path (black) shows the average positions for the whole sequence.

**Test #2**: Figure 9a shows the estimated positions of the human while walking following a linear path (red line) at a distance of Z = 1.2 m from the robot with X∈[−0.8,0.8] m, in both directions. The estimated path (black lines) diverges from the ground truth along the *z*-axis by approximately 10 cm. Since the employed detection model identifies the ankles (which are closer to the floor, but not touching it), this low error is always present but negligible due to its magnitude. This effect is also shown in Figure 8 as a constant bias in all the considered positions. The maximum error is localized at the endpoints of the trajectory in the *x*-axis (approximately 20 cm between the right foot’s real and estimated positions), as can be seen in the zoomed sections of the figure. This effect is also present in *Tests #1* and *#3*.

**Test #3**. Finally, Figure 9b represents the obtained poses for a human following a rectangular path of dimensions X×Z = 1.6 m × 0.8 m. Similar to *Test #2*, the main differences between the estimation and the ground truth paths are concentrated at the lateral sides.

**Figure 10:**
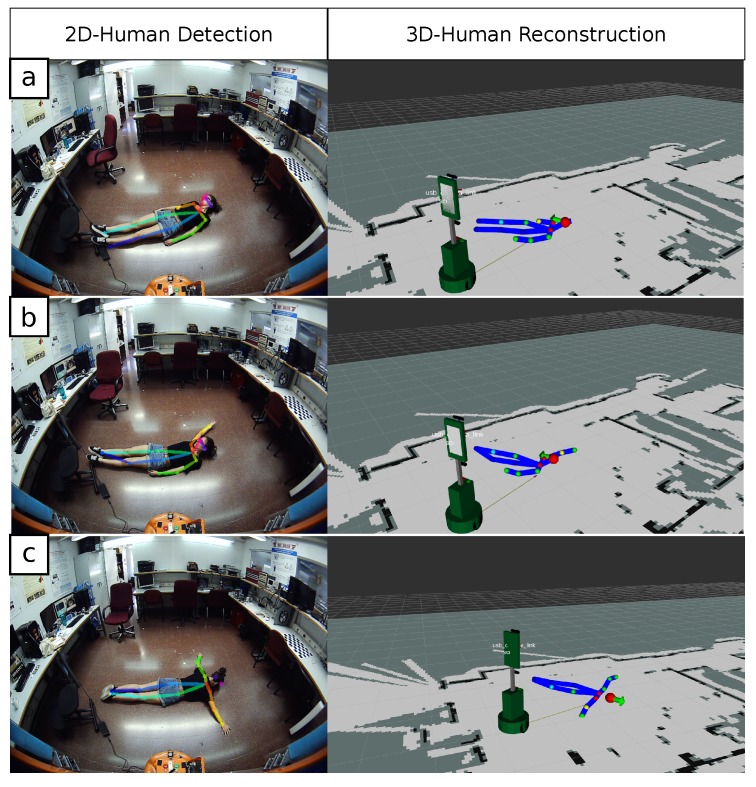
3D human bodies reconstructed with our *Single-View* approach with the user lying on the floor, (**a**) face-up, (**b**) face-up with extended arm, (**c**) face-down.

As a particular variation of the problem of detecting humans, our proposed system has the advantage of having the ability to detect and reconstruct humans lying on the floor, or any other horizontal surface. This skill acquires capital importance in fields like social robotics, where performing tasks such as fall detection of elderly people at home are becoming a prominent objective in research projects. To illustrate this, Figure 10a–c shows three human skeletons (including their orientation) reconstructed with the proposed system while the user is lying on the floor in front of the robot. While the *Orbbec Astra* camera is not able to detect the user in that posture, our proposal successfully locates them and estimates their 3D position in the environment. Considering the position of the torso joint as the center of the body, our system is capable of estimating their location. As explained in Section 4.1, the 3D position of the joints of a lying human are computed with the same formula employed for the triangulation of the feet of a standing person (Equation (3)), as employed in *Tests #1, #2,* and *#3* to compute the human’s position. Similar error values (below 0.2 m) are thus obtained with respect to the ground truth position, determined by a measuring tape.

To illustrate this scenario, we provide a video [49] recorded in both a laboratory environment and a house in which our *Single-View* approach is able to detect and estimate the position of a human body while lying on the floor. Finally, it has to be highlighted that, since the *Orbbec Astra* camera does not provide any result in this situation, a quantitative comparison with our proposal cannot be provided.

#### 5.2.2. Multi-View Method

Our *Multi-View* approach is mainly aimed to obtain robust estimations in difficult conditions regarding the human’s pose in the scene. Concretely, body parts occlusions, lost of joints during tracking or lack of information in the human orientation are overcome by this approach, proving its versatility when the *Single-View* approach finds difficulties to correctly determine the human’s body position in 3D.

**Figure 11:**
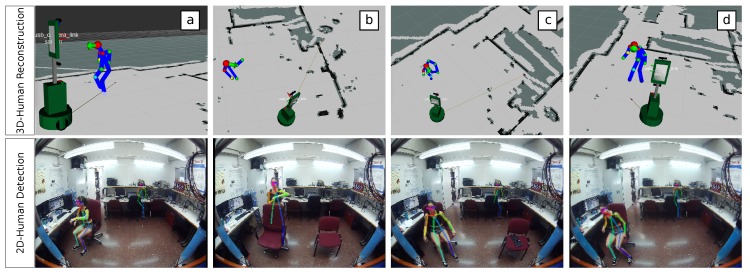
Human body reconstructions recovered with the *Multi-View* method under challenging situations: (**a**) sitting; (**b**) presence of occlusions; and (**c**,**d**) both sitting and near the camera.

This can be seen in Figure 11a, which shows a person who is detected and correctly located in 3D while sitting in front of the camera. Figure 11b illustrates a scenario where the person is occluded by some obstacle but our proposal still manages to infer their position Figure 11c,d shows similar scenarios to Figure 11a but this time with the person sitting quite close to the camera, hence being detected near the image border. This represents a really challenging situation for RGB-D cameras, as they present a minimum range of operation, typically above 0.5 m.

**Figure 12:**
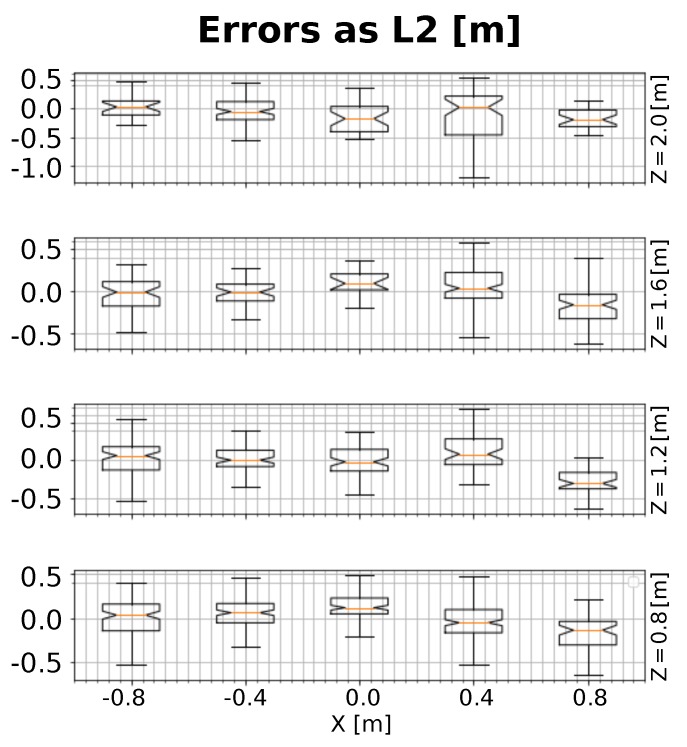
Average errors measured as L2-distances from the estimated positions to their corresponding ground truth with our *Multi-View* method.

Apart from the usability, and regarding the method’s accuracy, Figure 12 shows the estimated errors for the same ground truth positions described in *Test #1* of the *Single-View* approach. It can be seen that error values remain below 0.2 m for most of the considered positions, and that they are larger as the value of the *X* coordinate increases, similar to the effect in the *Single-View* method.

### 5.3. Human Orientation

**Figure 13:**
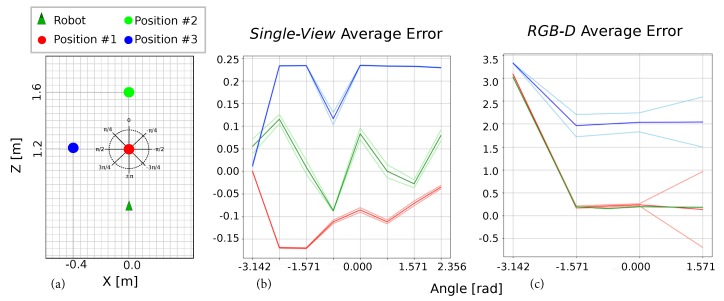
(**a**) Distribution of the positions and ground truth angles with respect to the robot frame; and (**b**,**c**) average error obtained for the proposed *Single-View* method and the RGB-D-based system, respectively.

Finally, to complete our evaluation, we focused on the accuracy of the human orientation estimated by our proposal and compared it again with that provided by the *Orbbec* SDK [39]. Figure 13 shows the results obtained from an experiment where the human adopted eight different orientations in [−π,π] with a π/4 step in three different positions [X,Z] (coordinates in meters): *Position #1*, [0.0,1.2]; *Position #2*, [0.0,1.6]; and *Position #3*, [−0.4,1.2].

In our method, the errors committed during the human detection stage are propagated to the orientation estimation so that body configurations where the torso is not completely or inaccurately discerned are prone to induce *oscillations* in the computed angle (e.g., ±π/2). However, even for challenging body configurations, errors are typically maintained below 0.25 rad.

In turn, for the RGB-D system, we directly extracted the head angle (and hence the user orientation) with its associated rotation matrix, which is provided by their SDK. No further processing was implemented. In this case, just four different angles were obtained: {0,−π/2,π/2,−π}, although some human bodily positions (e.g., with self-occlusions) dropped the detection rate and, consequently, the orientation estimation. As can be seen in the figure, *Position #3* presented the largest errors and the largest amount of loss of body tracking due to is closeness to its FoV limit. Additionally, we found that the distinction between the 0 and −π angles could not be distinguished in any of the considered positions, being impossible to tell if the user was facing towards the camera or not. Although a more accurate system could be investigated and built upon this 3D human body information provided by the RGB-D camera software, it goes beyond the scope of this work.

## 6. Conclusions and Future Work

In this study, we investigated the capabilities of wide FoV RGB cameras for 3D human pose estimation. This research arises with the aim of overcoming the inherent issues presented by RGB-D cameras, such as their narrow FoV, their short range of operation (under 3.5 m) and the strong influence of the light conditions (particularly sunlight). For that, we provided a mobile robot with the ability of first detecting a person while navigating and subsequently determining their poses (both position and orientation) within the environment, using as only input the images captured by a fish-eye webcam. Since the camera is attached to the robot’s tilting head and due to the specific characteristics of its movement, we also proposed a specific camera extrinsic calibration procedure.

This two-stage pipeline (detection and pose estimation) allows the robot to naturally navigate towards the user and establish an interaction. Additionally, our proposal is able to detect the human posture, indicating if they are standing up or lying down, hence providing semantic information that can be used to change the robot’s behavior, for instance, in terms of navigation.

For the first stage, we relied on the CNN-based person detector *OpenPose*, which provides the image coordinates of a set of detected joints. Built upon this detection system, we proposed two reconstruction methods:

- *Single-View*. It requires only a single view of the scene and allows the robot and the user to move during the pose estimation. Provided that the feet are detected, a simplified, planar human body is then reconstructed based on the camera pose and the feet coordinates, even determining if the user is standing up or lying down.
- *Multi-View*. It overcomes the *visible-feet* limitation by employing several views of the scene taken from different angles, requiring both the human and the robot to remain still during the process (≈2 s). Due to the particular camera setup and motion, common epipolar constraints for stereo vision can not be applied, thus we provided specific 3D reconstruction equations and integrated the body structure to improve its reconstruction.

The proposed methods were validated with both moving and static users in a laboratory environment, yielding similar accuracy results than an RGB-D camera-based system, while overcoming the above-mentioned limitations associated with them. Furthermore, we proved that our approach is able to both detect and locate the users even if they are lying on the floor, which cannot be accomplished by the *Orbbec Astra SDK* through human body tracking. This capability is of particular interest for social robots operating within elder’s homes for tasks such as fall detection.

We are currently integrating our work into social navigation systems for mobile robots in which humans and their personal areas are taken into account when planning the path to be followed by the robot. Face detection and recognition systems will also be combined with our proposal in order to allow the robot to distinguish between users before and during the interaction.
